# Modelling stunting in LiST: the effect of applying smoothing to linear growth data

**DOI:** 10.1186/s12889-017-4744-3

**Published:** 2017-11-07

**Authors:** Simon Cousens, Jamie Perin, Parul Christian, Lee Shu-Fune Wu, Sajid Soofi, Zulfiqar Bhutta, Claudio Lanata, Richard L. Guerrant, Aldo A. M. Lima, Kåre Mølbak, Palle Valentiner-Branth, William Checkley, Robert H. Gilman, R. Bradley Sack, Robert E. Black, Jean Humphrey, Neff Walker

**Affiliations:** 1 Department of Infectious Disease Epidemiology, London School of Hygiene and Tropical Medicine, London, UK; 2 Department of International Health, Johns Hopkins Bloomberg School of Public Health, Baltimore, MD USA; 3 The Bill & Melinda Gates Foundation, Seattle, WA USA; 4 Center of Excellence in Women and Child Health, the Aga Khan University, Karachi, Pakistan; 5 Centre for Global Child Health, the Hospital for Sick Children, Toronto, Canada; 6 Instituto de Investigación Nutricional, Lima, Peru; 7 Center for Global Health, University of Virginia School of Medicine, Charlottesville, VA USA; 8 Institute of Biomedicine, Faculty of Medicine, Federal University of Ceará, Fortaleza, Ceará Brazil; 9 Department of Infectious Diseases Epidemiology, Statens Serum Institut, Copenhagen, Denmark

**Keywords:** Lives saved tool, Mixed effects modelling, Nutritional interventions, Stunting

## Abstract

**Background:**

The Lives Saved Tool (LiST) is a widely used resource for evidence-based decision-making regarding health program scale-up in low- and middle-income countries. LiST estimates the impact of specified changes in intervention coverage on mortality and stunting among children under 5 years of age. We aimed to improve the estimates of the parameters in LiST that determine the rate at which the effects of interventions to prevent stunting attenuate as children get older.

**Methods:**

We identified datasets with serial measurements of children’s lengths or heights and used random effects models and restricted cubic splines to model the growth trajectories of children with at least six serial length/height measurements. We applied WHO growth standards to both measured and modelled (smoothed) lengths/heights to determine children’s stunting status at multiple ages (1, 6, 12, 24 months). We then calculated the odds ratios for the association of stunting at one age point with stunting at the next (“stunting-to-stunting ORs”) using both measured and smoothed data points. We ran analyses in LiST to compare the impact on intervention effect attenuation of using smoothed rather than measured stunting-to-stunting ORs.

**Results:**

A total of 21,786 children with 178,786 length/height measurements between them contributed to our analysis. The odds of stunting at a given age were strongly related to whether a child is stunted at an earlier age, using both measured and smoothed lengths/heights, although the relationship was stronger for smoothed than measured lengths/heights. Using smoothed lengths/heights, we estimated that children stunted at 1 month have 45 times the odds of being stunted at 6 months, with corresponding odds ratios of 362 for the period 6 to 12 months and 175 for the period 12 to 24 months. Using the odds ratios derived from the smoothed data in LiST resulted in a somewhat slower attenuation of intervention effects over time, but substantial attenuation was still observed in the LiST outputs. For example, in Mali the effect of effectively eliminating SGA births reduced prevalence of stunting at age 59 months from 44.4% to 43.7% when using odds ratios derived from measured lengths/heights and from 44.4% to 41.9% when using odds ratios derived from smoothed lengths/heights.

**Conclusions:**

Smoothing of children’s measured lengths/heights increased the strength of the association between stunting at a given age and stunting at an earlier age. Using odds ratios based on smoothed lengths/heights in LiST resulted in a small reduction in the attenuation of intervention effects with age and thus some increase in the estimated benefits, and may better reflect the true benefits of early nutritional interventions.

**Electronic supplementary material:**

The online version of this article (10.1186/s12889-017-4744-3) contains supplementary material, which is available to authorized users.

## Background

The Lives Saved Tool (LiST) is a freely available software package which allows users to explore the potential impact of scaling-up different interventions on a number of maternal and child health outcomes, notably mortality and nutritional (anthropometric) status. It is designed to help policy-makers and program managers in low- and middle-income settings make evidence-informed policy and investment decisions [1, 2].

In LiST, child stunting is both an outcome in its own right as well as a risk factor for child mortality. In 2013, the *Lancet* published a series on maternal and child nutrition which included an exercise using LiST to model the impact on child mortality of scaling up a range of nutrition-related interventions in the 34 countries that account for 90% of the world’s stunted children [3]. In the course of this exercise, it was noted that the effect on stunting of interventions during pregnancy to reduce the risk that a baby is born small-for-gestational-age (SGA) attenuated rapidly. For example, we noted that introducing a simulated intervention that effectively eliminated all SGA births reduced stunting prevalence at 1 month by 7.0%, but only reduced stunting prevalence at age 60 months by 1.5%. While some attenuation of effect is to be expected, there were concerns that the observed attenuation was greater than one might reasonably expect. We therefore sought to understand which aspects of the way in which nutrition outcomes are implemented in LiST might explain this rapid attenuation, and to investigate whether modifications to LiST’s approach to modelling stunting are required.

### How LiST models stunting

LiST is a population-based cohort model which predicts stunting rates from birth up to 5 years of age. For the sake of simplicity, it models children’s progress at the group level; that is to say, it models the prevalence of stunting in the population as a whole, and how this evolves with age, but does not track the progress of individual children. Also for simplicity, LiST works with discrete age bands, estimating the prevalence of stunting at the end of each age band (i.e., at 1, 6, 12, 24, and 60 months of age). Thus, for example, in LiST the prevalence of stunting at age 6 months influences but does not fix the prevalence of stunting at age 12 months (Table 1) under the assumption that some children who were stunted at 6 months may cease being stunted at 12 months, while other children who were not stunted at age 6 months may become stunted at 12 months. Note that this is a Markov model in the sense that the prevalence of stunting at, for example, 12 months depends on the prevalence of stunting at 6 months, but given the prevalence of stunting at 6 months is independent of the prevalence of stunting at earlier ages.

**Table 1:** Cross-tabulation of stunting status at 12 months versus stunting status at 6 months for a hypothetical population

		Status at 12 months	
		Stunted	Not stunted
Status at 6 months	Stunted	A	B
	Not stunted	C	D

### The phenomenon of decaying intervention effects

LiST allows for the possibility that, in the absence of any intervention, children who were stunted at, for example, 6 months cease to be stunted at 12 months, while other children who were not stunted at age 6 months become stunted at 12 months. Using the notation in Table 1, the extent to which children switch between being stunted and not being stunted can be quantified in terms of Ω = C/D (the odds of stunting at 12 months in those not stunted at 6 months) and B/A (the odds of not being stunted at 12 months for those stunted at 6 months). But B/A = 1/(Ω x R) where R is the “stunting-to-stunting” odds ratio (= AD/BC using the notation in Table 1). If stunting prevalence at both 6 and 12 months is fixed, then as R increases Ω gets smaller, and hence the amount of switching is reduced.

A consequence of this switching is that the effect of an intervention occurring in an early age band decays as children pass through subsequent age bands in the absence of any ongoing intervention. To demonstrate, suppose that currently the prevalence of stunting at 6 months is p_6_ and that the prevalence at 12 months is p_12_. Then the relationship between p_6_ and p_12_ is given by:

$$ {\mathrm{p}}_{12}=\left(1\hbox{-} {\mathrm{p}}_6\right)\times \Omega /\left(1+\Omega \right)+{\mathrm{p}}_6\times \Omega \mathrm{R}/\left(1+\Omega \mathrm{R}\right) $$

where Ω/(1 + Ω) is the probability that an unstunted child becomes stunted while ΩR/(1 + ΩR) is the probability that a stunted child remains stunted. Now suppose that we intervene prior to 6 months and reduce the prevalence of stunting at 6 months from p_6_ to p_6_*: i.e. stunting at 6 months is reduced by a factor p_6_*/p_6_. If we do not intervene after 6 months of age the prevalence of stunting at 12 months will be given by (replacing p_6_ with p_6_*):

$$ \left(1\hbox{-} {{\mathrm{p}}_6}^{\ast}\right)\times \Omega /\left(1+\Omega \right)+{{\mathrm{p}}_6}^{\ast}\times \Omega \mathrm{R}/\left(1+\Omega \mathrm{R}\right) $$

This means that stunting at 12 months will be reduced by a factor of

$$ \underline {\left(1\hbox{-} {{\mathrm{p}}_6}^{\ast}\right)\times \Omega /\left(1+\Omega \right)+{\mathrm{p}}_6\ast \times \Omega \mathrm{R}/\left(1+\Omega \mathrm{R}\right)} $$

$$ \left(1\hbox{-} {\mathrm{p}}_6\right)\times \Omega /\left(1+\Omega \right)+{\mathrm{p}}_6\times \Omega \mathrm{R}/\left(1+\Omega \mathrm{R}\right) $$

which is not equal to p_6_*/p_6_. A simple scenario illustrates this phenomenon. Without intervention, the prevalence of stunting is assumed to remain constant at 50% between 6 and 59 months of age. We also assume that the “stunting-to-stunting” odds ratio (R) takes the value 50 during each of the age bands. Under these assumptions, implementing an intervention prior to age 6 months which reduces the prevalence of stunting at 6 months from 50% to 25%, followed by no intervention after 6 months, will result in a reduction from 50% to 31% in stunting at age 12 months, from 50% to 35% at 24 months, and 50% to 39% at 60 months of age. In the version of LiST used for the 2013 *Lancet* series, the stunting-to-stunting ORs used were all less than 50, some much less than 50. Thus with the odds ratios currently used within LiST, the attenuation will actually be more extreme than the scenario illustrated above, that is, predicted stunting prevalence at 60 months of age will be closer to 50%.

Previous work presented in the Lancet Nutrition series (2013) was based on stunting-to-stunting odds ratios derived from measured lengths/heights in seven cohorts with data from 2375 children from four countries (see Table 2). These studies did not have sufficient data to estimate separately the association between stunting at 1 month and stunting at 6 months of age. A primary aim of this work was therefore to identify additional data to increase the sample size used to estimate these odds ratios.

**Table 2:** Data sources, numbers of children and numbers of length/height measurements contributing to the analysis

Data source		Number of children retained in the analysis	Number of records retained in the analysis
Pakistan MNP trial		2153	22,623
Nepal NNIPS-1		3723	24,830
Jivita (Bangladesh)		7873	47,238
Zvitambo (Zimbabwe)		3355	25,397
Cebu (Philippines)		2307	26,159
Multi-country database	Peru 1985^a^	519	7850
	Peru 1989^a^	192	2704
	Peru 1995^a^	224	5031
	Brazil 1989^a^	106	715
	Guinea-Bissau 1987^a^	397	3110
	Guinea-Bissau 1996^a^	684	7716
	Bangladesh 1993^a^	253	5408
Total		21,786	178,786

^a^Data sources contributing to the 2013 Lancet Nutrition series

### Potential for bias when estimating the stunting-to-stunting odds ratio

In addition to uncertainty arising from small sample sizes, a possible source of bias in the initial estimates of these odds ratios comes from measurement error. The stunting-to-stunting odds ratio is the key parameter determining the rate at which intervention effects attenuate over time. These ORs were derived from field studies that followed children over time, repeatedly measuring their length/height and classifying children as stunted or not stunted at 6, 12, etc. months of age. However, length/height measurements, particularly of very young children, are subject to measurement error. In epidemiological studies, random (non-differential) measurement error of both binary exposure and outcome is well known to result in estimated risk and odds ratios which are biased towards the null (1.0) [4]. Thus, random measurement error of length/height in the studies used by LiST will tend to result in an underestimate the stunting-to-stunting OR and hence an overestimate of the amount of switching in stunting status that occurs. For an intuitive explanation of why this will be so, consider an extreme scenario in which every child perfectly tracks a particular centile of the growth standard. In these circumstances, no children switch from being stunted to not stunted, or vice versa, and the true stunting-to-stunting OR is effectively infinite. Now suppose that children are measured with random error and their stunting status determined based on these error-prone measurements. Some children will appear to switch their status so that the estimated stunting-to-stunting odds ratio will be finite and thus biased towards the null compared with the true odds ratio.

In practice, relatively small measurement errors can have an important effect on a child’s estimated length/height-for-age z-score (LAZ/HAZ) and could therefore lead to substantial attenuation of the stunting-to-stunting OR. A measurement error of 0.5 cm in length/height will change a girl’s LAZ/HAZ by ± 0.10 at age 60 months, and by ± 0.16, ± 0.19, ± 0.22, and ± 0.26, at age 24, 12, 6, and 1 months, respectively. The aims of the work described in this paper were to:

1. Increase the pool of cohorts contributing to the estimation of stunting to stunting ORs.
2. Account for measurement error of lengths/heights when estimating stunting to stunting ORs.
3. Assess the effect of accounting for measurement error on estimates of stunting prevalence obtained from LiST.

## Methods

**Fig. 1:**
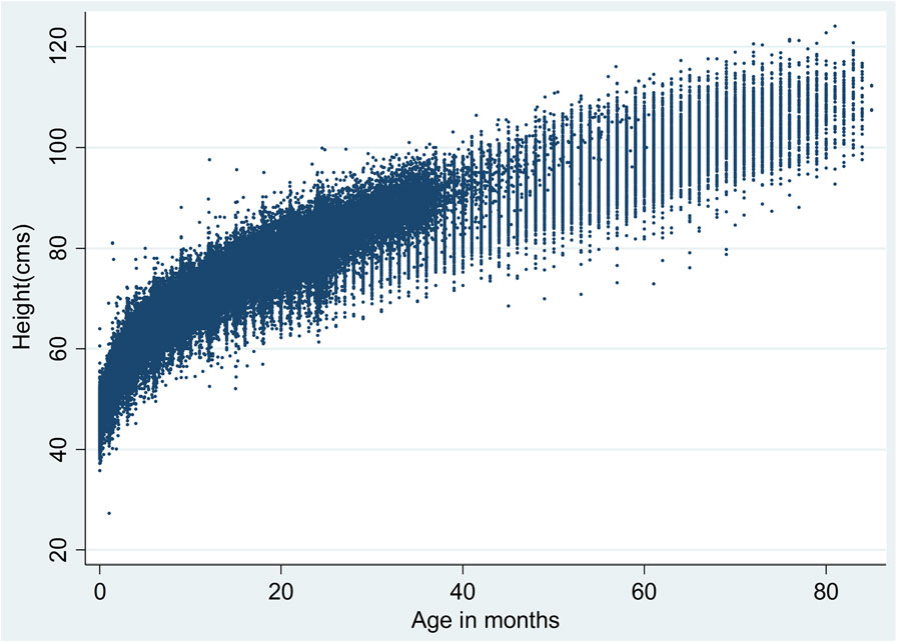
Distribution of measured lengths/heights by age for 21,786 children with at least six measurements between age 0 and 60 months among twelve cohorts

We identified datasets with serial measurements of length/height [5–10] (Table 2). Within each dataset we retained the records for children with at least 6 serial measurements. Figure 1 shows the distribution of measured lengths/heights for the 21,786 children retained in the analysis. Before fitting growth curves to the data, we applied the following transformation to age

$$ \mathrm{transformed}\ \mathrm{age}={\left(\mathrm{age}\ \mathrm{in}\  \mathrm{months}\right)}^{0.35} $$

This transformation resulted in a more linear relationship between (transformed) age and length/height (Additional file 1: Figure S1) and is the transformation used by the WHO Multicentre Growth Reference Study Group to produce the WHO Child Growth Standards (11). Restricted cubic splines (with knots at 1, 2, and 3 of transformed age) were used to model individual children’s growth curves, with random intercepts and coefficients for each child, using the mixed command in Stata 14 (www.stata.com). The model was fitted separately to each cohort, with the exception that we combined the Peru and Brazil cohorts from the multi-country study. Fitted (i.e. smoothed) lengths/heights were estimated taking account of both the fixed and random components of the model. LAZ/HAZs were then computed for measured lengths/heights and smoothed lengths/heights using the WHO reference standard [11].

Based on these z-scores, children were then classified as stunted/not stunted and stunting-to-stunting odds ratios were computed, where stunting was defined as LAZ/HAZ < −2. To combine ORs from different studies, we used the Mantel-Haenszel method [12]. After obtaining two sets of stunting-to-stunting ORs (based on measured and smoothed lengths/heights), each set was used in a LiST analysis to examine how quickly intervention effects attenuated. We also compared this attenuation to that estimated using the stunting-to-stunting odds ratios from the Lancet Nutrition series (2013). For this analysis we created baseline models based on two sub-Saharan African countries that have different birth outcome and stunting profiles, Mali in the year 2013 and the Republic of Congo for 2015. For the base year in Mali, we estimated that roughly 35% of children are born SGA, 44.4% of children aged 24-59 months are stunted. In the Republic of Congo, roughly 25% of children are born SGA and 25% are stunted between the ages of 24-59 months. To examine how the different sets of odds ratios affected attenuation, we ran an analysis in which we introduced a new intervention that is 99% effective in eliminating SGA births and scaled up coverage to 99% and examined the estimated impact on stunting prevalence at ages 1, 6, 12, 18, 24, and 60 months under each set of stunting-to-stunting ORs.

## Results

We identified and obtained data from five additional cohorts, increasing the number of children included in our analyses to 21,786. Three of the new studies included over 13,000 children observed close to 1 month of age, enabling us to estimate odds ratios from birth to 1 month, and from 1 month to 6 months.

**Fig. 2:**
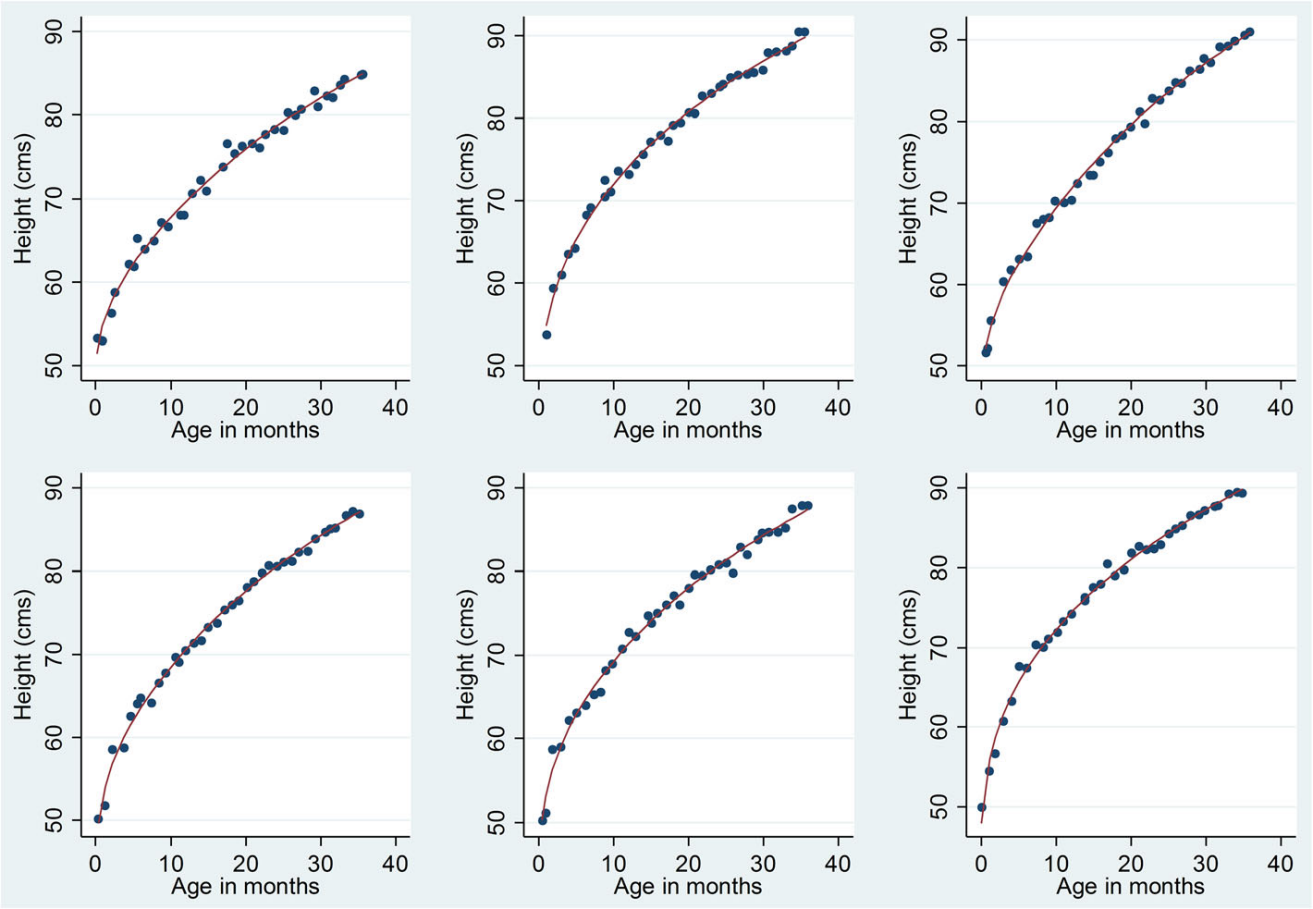
Some examples of fitted growth curves for individual children from the Pakistan data set

Figure 2 shows the results of the fitting procedure for 6 children from Pakistan. The figure illustrates the phenomenon of measurement error in that there are clearly some instances where a later length/height measurement is less than an earlier measurement. Assuming that children do not shrink in size this must reflect measurement error in one or both of the measurements. The smoothed growth curves appear to fit the measured data well.

While the individual fitted growth curves appear to track the measured lengths/heights reasonably well, there was some evidence of small systematic differences between measured and fitted lengths/heights at key ages. At 1 month of age, smoothed lengths were, on average, slightly greater than measured lengths (5 mm greater). At 6 months, fitted lengths were slightly shorter than measured lengths (2 mm shorter). At 12 months, the difference was about 3 mm (smoothed longer than measured). At 24 months, the difference was about 1 mm, but this time smoothed shorter than measured. At 60 months, the mean measured and fitted lengths were very similar. A comparison of measured and fitted lengths/heights across all cohorts is tabulated in Table 3.

**Table 3:** A comparison of measured and fitted lengths/heights at different ages

Age in months	Number of children	Mean measured length/height (cms)	Mean fitted length/height (cms)	Mean difference (95% c.i.)
1	10,028	52.2	52.7	0.48 (0.46, 0.51)
6	15,193	64.4	64.2	−0.22 (−0.24, −0.20)
12	15,094	71.1	71.4	0.26 (0.24, 0.28)
24	11,822	80.2	80.1	−0.09 (−0.11, −0.07)
60	1809	98.37	98.39	0.02 (−0.00, 0.04)

Table 4 presents the stunting-to-stunting ORs derived from the measured and smoothed lengths/heights. Smoothing led to a substantial increase in the stunting-to-stunting ORs for all age bands analysed. Odds ratios with measured lengths/heights were similar to those used in the 2013 Lancet Nutrition series (11 versus prior 12.4 for 1 to 6 months, 21 versus 21.4 for 6 to 12 months, and 22 versus 30.3 for 12 to 24 months), despite the additional cohorts.

**Table 4:** Stunting-to-stunting odds ratios estimated using measured and smoothed

	1 to 6 months		6 to 12 months		12 to 24 months	
Cohort	Measured	Smoothed	Measured	Smoothed	Measured	Smoothed
Pakistan	4	9	7	291	11	65
Nepal					Infinity	115
Peru/Brazil	11	5	47	522	20	39
Guinea-Bissau	36	25	31	337	19	223
Bangladesh	12	11	23	138	20	89
JIVITA	13	52	46	386	32	1770
Cebu			15	335	18	134
Zvitambo	8	47	14	348	18	82
Combined	11	45	21	362	22	175

Table 5 shows the results obtained from LiST when using different sets of stunting-to-stunting ORs. The simulated intervention almost eliminated SGA births in both countries. However, the impact of this reduction on later stunting varied depending on which set of stunting-to-stunting ORs were used. With the measured odds ratios, the virtual elimination of SGA births only reduced stunting prevalence at age 60 months in children from 44.4% to 43.7% (a 1.6% reduction) in Mali and from 25.1% to 24.8% (a 1.2% reduction) in the Republic of Congo. In the scenario using the smoothed odds ratios, the stunting prevalence among children aged 60 months in Mali were reduced from 44.4% to 41.9%, a 5.6% reduction in stunting and in the Republic of Congo stunting in this age group dropped from 25.1% to 24.0%, a 4.4% reduction in stunting. As expected, the use of the smoothed ORs reduced the attenuation effects seen with the measured odds ratios.

**Table 5:** Results from a LiST analysis using different stunting to stunting ORs

	Stunting-to-stunting OR type used	Prevalence of SGA at birth (%)	Prevalence of stunting at age (%)				
			1 month	6 months	12 months	24 months	60 months
*Mali*							
No intervention	–	35.2	14.9	14.9	17.2	37.3	44.4
Intervention	Prior ORs^a^	0.7	8.1	11.9	15.4	36.1	43.5
	Measured	0.7	8.1	12.0	15.5	36.2	43.7
	Smoothed	0.7	8.1	10.4	13.2	34.3	41.9
*Republic of Congo*							
No intervention	–	24.6	8.5	8.5	15.3	29.6	25.1
Intervention	Prior ORs^a^	0.5	5.0	7.3	14.6	29.1	24.8
	Measured	0.5	5.0	7.3	14.6	29.2	24.8
	Smoothed	0.5	5.0	6.5	13.5	28.2	24.0

^a^From 2013 Lancet Nutrition series

## Discussion

In this paper we presented analyses of child growth trajectories to estimate associations between stunting at different ages. We increased substantially the amount of information contributing to these estimates compared with previous analyses. The number of contributing children increased from 2375 to 21,785, the number of individual length/height measurements increased from 32,534 to 178,787, and the number of countries represented increased from 4 to 8. This increase in the data available for estimating the ORs resulted in a more robust set of estimates of the ORs between age periods, providing more precise parameter estimates for use in the model. In addition to increased data inputs, we applied smoothing to eliminate or reduce measurement error in recorded lengths/heights and examined the effect of this smoothing on results obtained from the LiST model.

Precisely measuring the length/height of very young children is challenging. Random errors in sequential measurements of length/height will tend to exaggerate variation in individual children’s LAZ/HAZs and thus the extent to which children switch from being stunted to not stunted and vice versa. The extent of such switching is a key parameter in LiST, with intervention effects attenuating more rapidly over time as the rate of switching increases. In the past, stunting was modelled in LiST using stunting-to-stunting ORs estimated from measured lengths/heights.

To investigate the extent to which measurement error may be affecting the results obtained from LiST, we used length/height measurements from over 20,000 children with at least six measurements each from birth to age 5 years, from cohorts representing several regions and settings where stunting is most common. We modelled individual children’s length/height over time using random effects models to produce smoothed growth trajectories for each child, in an attempt to remove random fluctuations in recorded measurements due to measurement error.

As expected, smoothing resulted in increases in the estimated stunting-to-stunting ORs in all age bands. Some of the ORs based on the smoothed lengths/heights were very large indeed. For example, the smoothed OR for the age band 6 to 12 months was approximately 360. ORs this large may strike readers more familiar with exposure-disease associations as implausibly large. In the stunting context, however, this represents a situation in which the proportion of children flipping between one status and the other is in the region of 5%. As the proportion of children who switch status decreases, the stunting-to-stunting OR increased, very rapidly as the switching rate falls below 10%. In the context of stunting switching rates of 10% or less may not be implausible, as it is often argued that once children become stunted they remain stunted [7].

The stunting-to-stunting ORs used in LiST will affect the predictions that the model produces with respect to the impact of interventions on stunting and, by extension, child mortality. The use of inappropriately small stunting-to-stunting ORs will result in the attenuation of intervention effects, especially those during pregnancy and the first few months of life. Using ORs based on measured lengths/heights, a simulated intervention that essentially eliminated SGA births only resulted in a tiny reduction in stunting prevalence at 60 months of age in Mali (from 44.4% to 43.7%). The effect of the same reduction in SGA births using stunting-to-stunting ORs based on smoothed lengths/heights resulted in a reduction in stunting at 60 months from 44.4% to 41.9%, a relative reduction of 5.6% in the prevalence of stunting. Unfortunately it is not known to what extent stunting is accounted for or explained by SGA, and thus there is no bench mark to measure this attenuation, even though SGA and stunting are strongly associated in some settings [13].

It might be argued that the smoothing procedure we used not only removes random fluctuations due to measurement error but also real variations in growth trajectories. However, even using the much larger ORs based on smoothed lengths/heights, there is still considerable effect attenuation by 60 months of age, with LiST outputs rather insensitive to even large changes in the stunting-to-stunting ORs. Any over-smoothing which did occur is therefore likely to have had only a very small effect on LiST outputs. Perhaps we should not be very surprised that an intervention effectively eliminating SGA at birth has limited impact on stunting at age 60 months, given the many factors contributing to stunting after a child is born and as they grow older.

There are some limitations to our analysis. We were not able to locate as many repeated measurements of length/height in children across the age range 24 to 59 months as for younger children, as the observations for the cohorts in this analysis were largely restricted to the period from birth to 2 years. Smoothing measured lengths/heights may be more appropriate for these younger children, if they are more difficult to measure and thus their measured lengths are more prone to error. Analysis of the association between stunting at different ages for older children is the object of future research for which we plan to seek further data.

A second limitation of our analyses is that we observed some small but systematic differences between average measured and smoothed lengths/heights, as shown in Additional file 2: Figure S2. The alternating direction of these differences suggests that the cubic spline functions that we fitted may not have been quite flexible enough to capture fully individual children’s growth trajectories.

## Conclusions

LiST is a powerful tool for predicting the health consequences of different interventions for young children in different low- and middle-income settings. Based on the results of our modelling, the stunting-to-stunting ORs used in LiST have been updated and LiST now uses the ORs derived from smoothed lengths/heights. While this has not resulted in major changes in the outputs obtained from LiST, we believe that accounting for measurement error is appropriate in this context and that predictions in LiST now reflect better the nature of stunting, its persistence in individual children, and the consequent benefit of early intervention for the health and growth of children under five. We note, however, that smoothing will not be appropriate in all circumstances: for example, it may not be appropriate in randomised trials to test the effectiveness of interventions.

## Additional files

Additional file 1: Figure S1. Distribution of measured lengths/heights against transformed age for 21786 children. (DOCX 1363 kb)

Additional file 2: Figure S2. A comparison of LAZ/HAZ scores based on measured and fitted lengths/heights for children aged 1, 6, 12, and 24 months. (DOCX 433 kb)

## Abbreviations

LAZ/HAZ: Length/height-for-age z-score
LiST: Lives saved tool
OR: Odds ratio
SGA: Small-for-gestational-age
WHO: World Health Organization
