# Supplementary material for: Modelling stunting in LiST: the effect of applying smoothing to linear growth data
Source: BMC Public Health. 2017 Nov 7;17(Suppl 4):778. doi: 10.1186/s12889-017-4744-3 (PMC5688407; doi:10.1186/s12889-017-4744-3)
Supplement: Supplementary file 1 — Distribution of measured lengths/heights against transformed age for 21786 children. (DOCX 1363 kb) [file 12889_2017_4744_MOESM1_ESM.docx]

**Supplemental Figure 1.** Distribution of measured lengths/heights against transformed age for 21786 children
