# Supplementary material for: Modelling stunting in LiST: the effect of applying smoothing to linear growth data
Source: BMC Public Health. 2017 Nov 7;17(Suppl 4):778. doi: 10.1186/s12889-017-4744-3 (PMC5688407; doi:10.1186/s12889-017-4744-3)
Supplement: Supplementary file 2 — A comparison of LAZ/HAZ scores based on measured and fitted lengths/heights for children aged 1, 6, 12, and 24 months. (DOCX 433 kb) [file 12889_2017_4744_MOESM2_ESM.docx]

**Supplemental Figure 2.** A comparison of LAZ/HAZ scores based on measured and fitted lengths/heights for children aged 1, 6, 12, and 24 months. The red lines indicate where smoothed and observed LAZ/HAZ are the same.
